# Association of global sagittal spinal deformity with functional disability two years after total hip arthroplasty

**DOI:** 10.1186/s12891-021-04415-1

**Published:** 2021-06-07

**Authors:** Yoshinori Okamoto, Hitoshi Wakama, Tomohiro Okayoshi, Shuhei Otsuki, Masashi Neo

**Affiliations:** Department of Orthopedic Surgery, Osaka Medical and Pharmaceutical University (Osaka Medical College), 2-7 Daigaku-machi, Takatsuki, Osaka, 5698686 Japan

**Keywords:** Disability, Global sagittal deformity, Spinopelvic alignment, T1 pelvic angle, Total hip arthroplasty

## Abstract

**Background:**

The relationship between spinopelvic alignment and functional disability after total hip arthroplasty (THA) has not been fully elucidated despite the growing recognition of its importance on patient-reported outcome measures. Therefore, our aim was to assess the effect of global sagittal spinal deformity on post-operative disability.

**Methods:**

This analysis was based on 208 cases of THA, with functional disability measured at a follow-up of 2 years. The Hip Disability and Osteoarthritis Outcome Score-Joint Replacement (HOOS-JR), ranging from a scale of 0 (complete joint disability) to 100 (perfect joint health), was used to divide eligible patients into two groups, namely *with* and *without* disability, using a score of 70 as the cut-off. The following factors were compared between the two groups using multivariate analysis: age, sex, body height, body mass index, spinopelvic parameters, and surgeon experience. To identify the cut-off value of the parameters for predicting disability (HOOS-JR < 70/100), we used the receiver-operating characteristic curve.

**Results:**

The disability (30 hips) and control (178 hips) groups showed a significant difference in pre-operative body height (*p* = 0.020), T1 pelvic angle divided by pelvic incidence (T1PA/PI; *p* = 0.018), PI minus lumbar lordosis (*p* = 0.027), post-operative HOOS-JR (*p* = 0.010), patient satisfaction (*p* = 0.033), and the modified Harris Hip Score (*p* = 0.038). On multivariate analysis, the following factors were associated with persistent disability: T1PA/PI > 0.2 (odds ratio [OR], 2.11; 95% confidence interval [CI], 1.19–4.14; *p* <  0.001) and height < 148 cm equivalent to legal standards as short stature (OR, 1.26; 95% CI, 1.09–1.48; *p* = 0.011). The cut-off value of pre-operative T1PA/PI was > 0.19, with a sensitivity of 95% and specificity of 85%. Post-operative satisfaction (*p* <  0.001), HOOS-JR (*p* = 0.023), and EuroQol 5-Dimension (*p* = 0.041) differed between the two groups when the pre-operative cut-off value was chosen as 0.2.

**Conclusions:**

A T1PA/PI > 0.2 was associated with greater disability after THA. Clinicians should be aware that patient-related factors, including global spinal deformities, particularly in patients with a short stature, can influence THA outcomes at 2 years postoperatively.

## Background

Despite the proven efficacy of total hip arthroplasty (THA), one in 7–14 patients still report persistent dissatisfaction on short- to medium-term follow-up [1–3]. Some studies have identified factors which influence patient satisfaction or functional disability after THA, such as pre-operative patient expectations, the degree of improvement achieved, mental health status, comorbidities, and pain relief [1, 2, 4]. Although patient satisfaction plays an important role in assessing therapeutic effects, the impact of the pre-operative spinopelvic alignment on disability after THA has not been reported, even if only over a short term.

Pertinent issues have been raised about the increased incidence of concurrent hip osteoarthritis (OA) and spinal deformities in aging populations [4], with spinal deformities identified in approximately 20–44% of patients undergoing THA [5, 6]. A greater understanding of the association between sagittal spinopelvic alignment and outcomes is also thought to minimise instances of cumbersome THA dislocation or revision [7, 8]. However, little is known about how sagittal spinal alignment affects THA outcomes, especially patient functional disability after THA [4, 9–11]. The key to successful THA necessitates a further comprehensive analysis of the influence of sagittal spinopelvic interactions on functional outcomes. This argument is important to evaluate considering the importance of patient-reported outcome measures (PROMs) in today’s healthcare system.

Furthermore, most large databases, such as the national joint registry or multi-centre studies, are limited to the analysis of PROMs, implant longevity, or complications [12–17]. However, no study has investigated the relationship between spinopelvic alignment and patient disability after THA. A better understanding of patient-related factors is essential to improve the prognosis of THA. Of these factors, resolving the controversy regarding the concurrence of sagittal spinal imbalance and hip OA for clinicians, patients, and policymakers would be particularly important, considering the general super-ageing of our society. Accordingly, the purpose of our study was to determine whether global sagittal spinal deformity is associated with functional disability after THA.

## Methods

### Participants

**Fig. 1:**
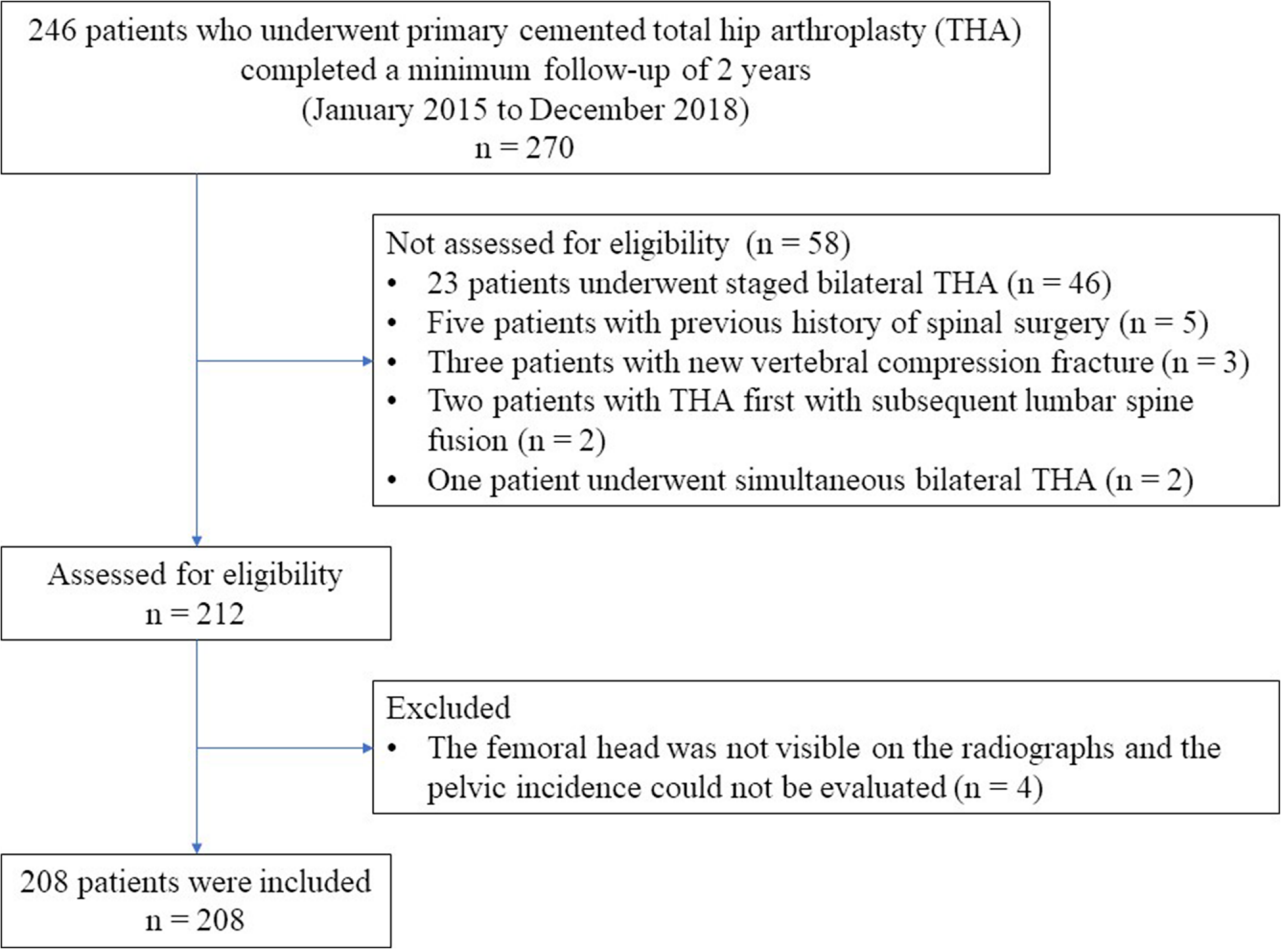
Flowchart of patient inclusion and exclusion criteria

The study was approved by the institutional review board of our hospital (approval number 1912) and performed in line with the principles of the Declaration of Helsinki (1964) and its subsequent amendments. All patients provided written informed consent for their participation in the study and the publication of their data. Between January 2015 and December 2018, 285 primary THAs were performed at our institution. Of these, 246 patients (270 hips), who were Asian, completed a minimum follow-up of 2 years and were enrolled into this study. From this group, we excluded 34 patients (58 hips) who had undergone a staged bilateral THA history (46 hips) or had a history of spinal surgery (five hips), new vertebral compression fracture (three hips) [18], THA with subsequent lumbar spine fusion (two hips), or simultaneous THA (two hips) during the follow-up period. For a few patients, the femoral head was not visible on radiographs and, thus, the pelvic incidence (PI) could not be evaluated (four hips) [19]. Ultimately, 208 patients (208 hips) were included in our study (Fig. 1). Of these, secondary hip OA was the most common (165 hips, 79.3%), followed by primary OA and osteonecrosis of the femoral head (18 hips for each, 8.7%) [20].

### Surgical procedure and post-operative protocol

All THAs were performed by six experienced arthroplasty surgeons using a direct lateral approach with the patient in the lateral decubitus position [21, 22]. Of these, 130 required acetabular structural bone grafting for the dysplastic acetabulum [22]. The highly cross-linked polyethylene flanged socket (K-MAX CLHO flanged cup, Kyocera Medical, Osaka, Japan) and a cobalt-chromium head with a polished stem (SC stem, Kyocera Medical, Osaka, Japan) were fixed using bone cement (CMW Endurance, DePuy, Blackpool, UK). All patients were allowed full weight-bearing post-operatively, with the use of crutches encouraged, as needed, for the first 3 months. This was according to a standardised fast-track post-THA protocol, which included standardised physical therapy with mobilisation after drain removal.

### Clinical evaluations

Before and at 2 years after THA, we used the modified Harris Hip Score (HHS) and the Trendelenburg sign as measures of hip function [23, 24]. The incidence of complications was investigated. Data were analysed in a blinded fashion.

### Patient-reported outcome measures

We evaluated the patient-reported outcomes pre-operatively and at 2 years post-operatively. The Hip disability and Osteoarthritis Outcome Score, Joint Replacement (HOOS-JR) is a short PROM developed to efficiently evaluate end-stage hip OA in patients undergoing THA. The HOOS-JR is a six-question survey derived from the original 40-question HOOS. Each item on the HOOS-JR is scored from 0 to 28 and then converted into an interval score from 0 (total joint related disability) to 100 (perfect joint health) [24, 25]. A 100-mm visual analogue scale (VAS) was used to evaluate hip pain and patient satisfaction. The 100-mm VAS-pain and satisfaction score was categorised for analysis from a range of “0” mm (no pain and very satisfied) to “100” mm (worst pain imaginable and completely dissatisfied) [15, 24]. The EuroQol 5-Dimension 5-Level (EQ-5D) scale was used as a measure of patient-reported quality of life [24, 26].

### Radiological evaluations

**Fig. 2:**
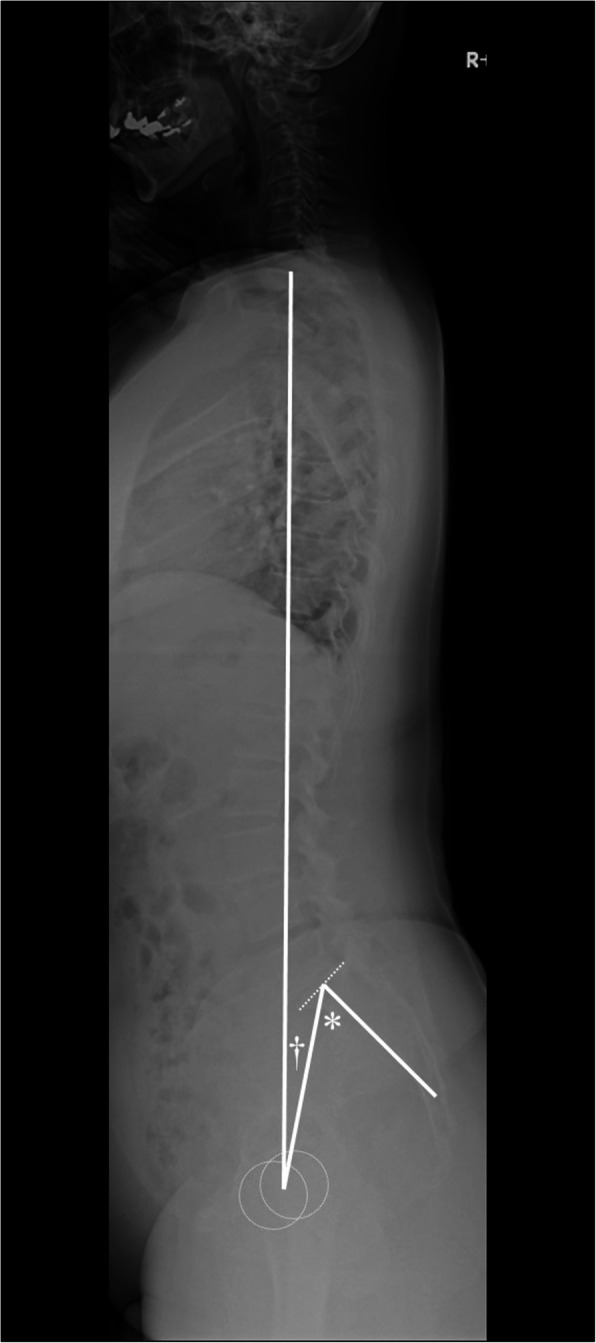
A radiograph was used to evaluate the pelvic incidence (*) and T1 pelvic angle (T1PA, †) of a patient with a global sagittal spinal deformity [6, 19]. T1PA is defined as the acute angle by the intersection of a line from the centre of the T1 vertebral body to the femoral heads and a line from the femoral heads to the centre of the superior sacral end plate. This interplay suggests useful information from both the sagittal vertical axis and pelvic tilt simultaneously to measure the geometry of global spinal deformity

Spinopelvic alignment was assessed before and at 2 years after THA, with the patients in the standing position [27]. Radiographs obtained within 1 month pre-operatively were reviewed for vertebral fractures by an independent arthroplasty surgeon with 10 years of experience. Vertebral fractures were identified using a semiquantitative method, namely a decrease in the height of the vertebral body > 20% [18]. Radiological measures of the sagittal spinopelvic alignment were obtained using a protractor with 1° increments as follows: C7 sagittal vertical axis (SVA), lumbar lordosis (LL), PI, pelvic tilt (PT), and T1 pelvic angle (T1PA) [4, 6, 19, 23, 28] (Fig. 2). The T1PA, a measure of the global malalignment and/or compensation through pelvic retroversion, was defined as the angle between the line from the femoral head axis to the centre of the T1 vertebral body and the line from the femoral head to the centre of the S1 superior end plate. A T1PA divided by PI (T1PA/PI) > 0.2, which provides an angular measure of global sagittal spinal deformity, was associated with lower health-related quality of life in patients undergoing treatment for adult spinal deformity [6]. Osseous complications at the reattached fragment were evaluated on anterior-posterior radiographs obtained 2 years after THA [21, 22].

To calculate the reliability of the spinopelvic alignment, three experienced arthroplasty surgeons independently evaluated the radiographic parameters, with each observer completing three randomly selected measurements at a mean interval of 4.1 (range, 3.6 to 4.4) weeks for 15 patients each. All observers were orthopaedic surgery specialists, with > 6 years of experience. Additionally, they had at least completed a 1-year fellowship in hip surgery under a mentor. Intra- and inter-rater reliability was calculated with a tolerance error of < 2° [29].

### Statistical analysis

Statistical analyses were performed using JMP 14 software (SAS Institute Inc., Cary, NC, USA), with *p*-values < 0.05 considered statistically significant. We defined a HOOS-JR of 70 as a clinically significant cut-off value and divided patients into the following two groups for comparison: the disability group, who had a post-operative HOOS-JR < 70, indicating hip disability, and the control group, who had a HOOS-JR ≥70, indicating no disability [30].

Differences in the measured variables between the two groups were evaluated using the Mann-Whitney U test for continuous variables. Categorical variables were compared using Fisher’s exact or chi-squared tests as per the data distribution. The Steel-Dwass test was used to reveal the relationship between the grade of OA according to the Kellgren-Lawrence classification and spinopelvic parameters. To identify independent risk factors for the residual disability group, logistic regression analyses were performed. Factors, such as age, sex, body height, body mass index, spinopelvic parameters, and surgeon experience, were analysed using an exploratory univariate analysis followed by a multivariate analysis [1, 4, 7, 13, 15–17, 22]. Short stature was typically defined as a height < 147.23 cm by legal standards [13]. Surgeons were classified into the following groups: orthopaedic specialists < 8 years’, 8–15 years’, and ≥ 15 years’ experience after certification [15].

A multicollinearity test was performed with the variance inflation factor set at < 10. Age was included as a confounding factor. To identify the cut-off value of the parameters for predicting disability, we used the receiver-operating characteristic (ROC) curve.

## Results

**Fig. 3:**
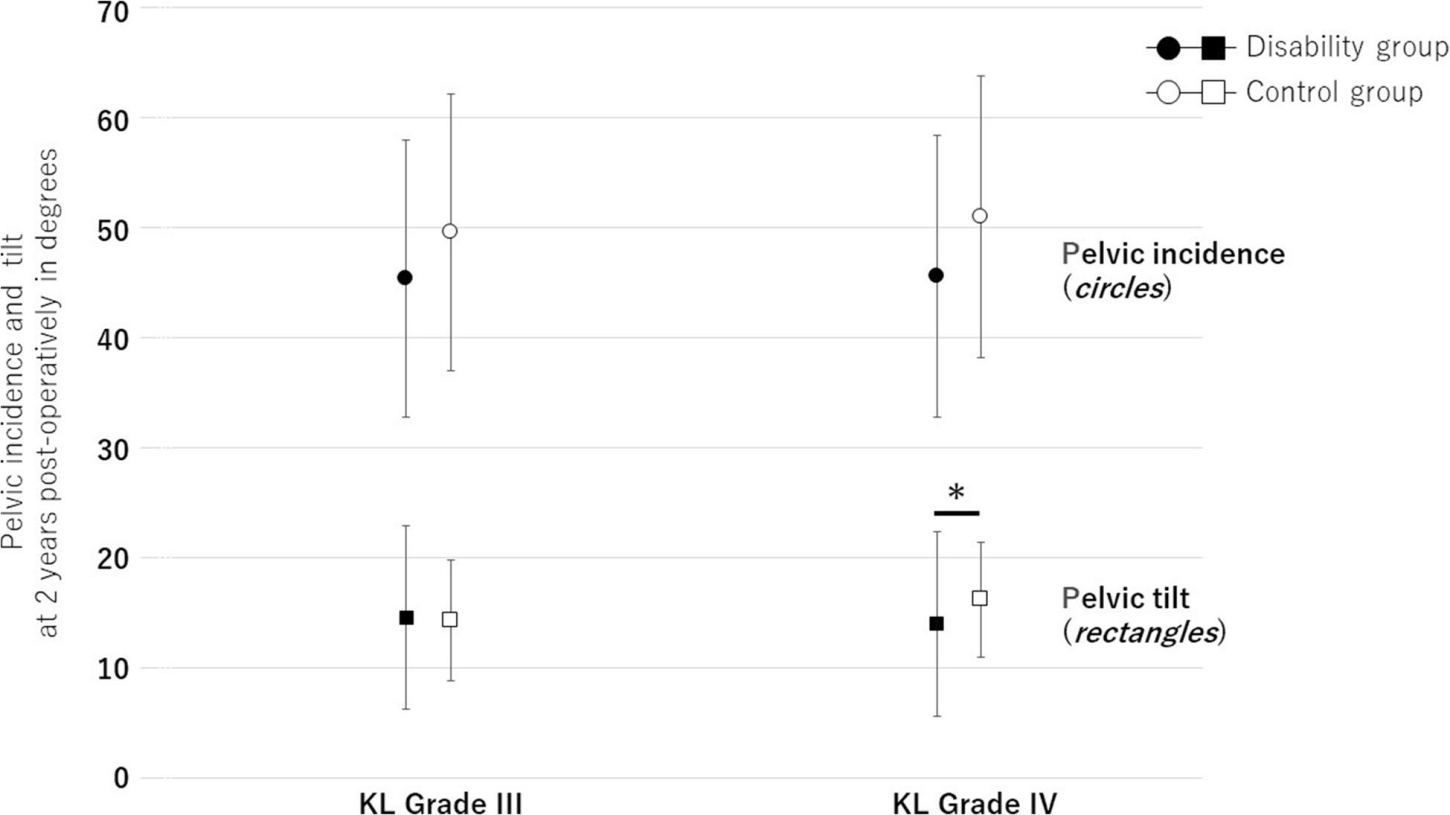
The relationship between pelvic incidence *(circles)* and pelvic tilt *(rectangles)* at 2 years post-operatively in degrees in patients classified as Kellgren-Lawrence (KL) grade III and IV. A statistically significant difference was seen between the disability and control groups (mean [*circles* or *rectangles*] ± standard deviation [error bar]) with regard to pelvic tilt in patients with KL grade IV (14.1° ± 8.5 and 16.2° ± 5.2, *p* = 0.034), although there was no difference in the measured values due to the radiographic severity. *P < 0.05; represents significant between-group differences

The disability (30 hips, 14.4%) and control (178 hips, 85.6%) groups showed a significant difference in body height (*p* = 0.020), pre-operative T1PA/PI (*p* = 0.018), PI minus LL (*p* = 0.027), post-operative Trendelenburg sign (*p* <  0.001), osseous complications (*p* = 0.006), HOOS-JR (*p* = 0.010), satisfaction (*p* = 0.033), and modified HHS (*p* = 0.038). With respect to LL and T1PA, the differences between the two groups were significant both preoperatively (*p* = 0.016 and *p* = 0.041, respectively) and postoperatively (*p* = 0.018 and *p* = 0.041, respectively) (Table 1). However, no significant differences were found between the two groups in terms of complications. Deep infections (one hip, 0.6%), peri-prosthetic femoral fractures (two hips, 1.1%), and post-operative dislocation (one hip, 0.6%) were observed in the control group, whereas dislocation, fracture, infections, and permanent sciatic nerve palsy (one hip for each, 3.3%) occurred in the disability group. In patients classified as Kellgren-Lawrence grade III, no differences between the groups were seen with respect to PI or PT postoperatively; however, PT in grade IV differed significantly (*p* = 0.034) (Fig. 3).

**Table 1:** Baseline characteristics and comparison of the disability and control groups

	Disability group *n* = 30	Control group *n* = 178	*P* value
Age (years)	74.9 ± 7.1	74.2 ± 7.3	0.745
Male (n, *%*)	4, *13.3*	21, *11.8*	0.811
Body height (cm)	153.1 ± 3.1	156.7 ± 4.3	0.020*
Body height < 148 cm (n, *%*)	6, *20.0*	12, *6.7*	0.042*
Body mass index (kg/m^2^)	25.3 ± 3.1	24.5 ± 3.1	0.723
Pre-operative diagnosis (n, *%*)			0.174
Primary osteoarthritis^a^	3, *10.0*	15, *8.4*	
Secondary osteoarthritis^a^	22, *73.3*	143, *80.3*	
Osteonecrosis of the femoral head	2, 6.7	16, *9.0*	
Others	3, *10.0*	4, *2.2*	
Kellgren-Lawrence classification (n)			
Garde II: III: IV	0: 5: 20	0: 32: 126	0.977
Prevalent vertebral fractures (n, *%*)			0.466
0	22, *73.3*	144, *80.9*	
1	6, *20.0*	29, *16.3*	
2+	2, *6.7*	5, *2.8*	
Surgeons’ experience (n, *%*)			0.310
< 8 years	2, *6.7*	6, *3.4*	
8–15 years	16, *53.3*	76, *42.7*	
> 15 years	12, *40.0*	96, *53.9*	
Modified Harris Hip Score^b, c^	49.2 ± 12.8	53.4 ± 13.1	0.544
	66.2 ± 7.8	91.7 ± 6.9	0.038*
Visual analogue scale-pain (mm)^c^	87.6 ± 21.8	71.8 ± 22.9	0.352
	22.7 ± 13.2	16.9 ± 13.1	0.611
Visual analogue scale-satisfaction (mm)^c^	84.1 ± 14.3	75.1 ± 13.7	0.747
	55.9 ± 12.3	14.9 ± 13.1	0.033*
HOOS-JR^c^	48.1 ± 13.8	45.9 ± 13.6	0.917
	52.8 ± 16.1	87.5 ± 14.3	0.010*
EuroQol 5-Dimension^c^	0.43 ± 0.17	0.43 ± 0.11	0.894
	0.63 ± 0.12	0.70 ± 0.12	0.125
Trendelenburg sign (n, *%*)^c^	8, *26.7*	43, *24.2*	0.768
	6, *20.0*	7, *3.9*	< 0.001*
C7 sagittal vertical axis (mm)^c^	25.1 ± 31.9	22.8 ± 33.5	0.763
	27.0 ± 31.7	25.2 ± 32.9	0.648
Lumbar lordosis (°)^c^	34.1 ± 15.5	52.9 ± 16.7	0.016*
	37.1 ± 14.8	50.5 ± 17.5	0.018*
Pelvic incidence (°)^c^	45.8 ± 13.2	47.2 ± 13.7	0.691
	45.6 ± 13.5	47.2 ± 13.4	0.863
Pelvic incidence minus lumbar lordosis (°)^c^	11.7 ± 11.7	−5.7 ± 10.7	0.027*
	8.5 ± 12.1	−3.3 ± 10.1	0.265
Pelvic tilt (°)^c^	13.7 ± 8.1	13.2 ± 10.8	0.724
	14.2 ± 9.3	14.8 ± 11.3	0.717
T1 pelvic angle (°)^c^	14.7 ± 13.2	8.5 ± 8.1	0.041*
	15.5 ± 13.8	9.9 ± 9.4	0.032*
T1 pelvic angle divided by pelvic incidence^c^	0.32 ± 0.11	0.18 ± 0.09	0.018*
	0.34 ± 0.14	0.21 ± 0.08	0.023*
Complication at the reattached fragment^d^			
Total, (n, %)	7, *23.3*	13, *7.3*	0.006*
Type I: II: III (n)	2: 3: 2	8: 4: 1	0.286

Data are expressed as mean ± standard deviation or the number of hip involvement (*%*) as appropriate for the data type

*P < .05; represents significant between-group differences

*HOOS-JR* the Hip Disability and Osteoarthritis Outcome Score Joint Replacement

^a^Primary hip osteoarthritis was unassociated with developmental dysplasia of the hip. The dysplasia was defined as a center edge angle of ≤25°, acetabular roof obliquity of ≥10°, or acetabular head index of ≤75% [20]

^b^Composite measure covering pain and function, scored on a scale ranging from 0 to 100, with a higher value representing improved function and decreased pain

^c^Between-group comparisons of outcomes pre-operatively (upper row) and at the two-year follow-up (lower row)

^d^Tip and base fractures of the greater trochanter for Types I and II, respectively; and a migration of the osteotomized fragment for Type III [22]

On regression analysis, patient age at the time of surgery was associated with neither pre-operative nor post-operative measures. The independent variables associated with greater disability were a T1PA/PI > 0.2 (versus a T1PA/PI ≤0.2; odds ratio, 2.11; *p* <  0.001) and body height < 148 cm (versus a height ≥ 148 cm; odds ratio, 1.26; *p* = 0.011) (Table 2).

**Table 2:** Univariate and multivariate logistic regression analyses of the risk factors for persistent disability after total hip arthroplasty defined by the Hip Disability and Osteoarthritis Outcome Score Joint Replacement < 70/100

	Univariate analysis	Multivariate analysis		
	*P* value	Odds ratio	95% CI	*P* value
Age	.684			
Male	> .999			
Body height				
≥ 148 cm	Reference			
< 148 cm	.078*	1.26	1.09–1.48	0.011**
Body mass index				
≥ 25	Reference			
< 25	.746			
Surgeons’ experience				
> 15 years	Reference			
8 to 15 years	> .999			
< 8 years	.174*	1.18	0.82–1.66	0.585
T1PA/PI				
≤ 0.2	Reference			
> 0.2	.012*	2.11	1.19–4.14	< 0.001**

*CI* confidence interval; *T1PA/PI* T1 pelvic angle divided by pelvic incidence

*P < .2, statistically significant

**P < .05, statistically significant

**Fig. 4:**
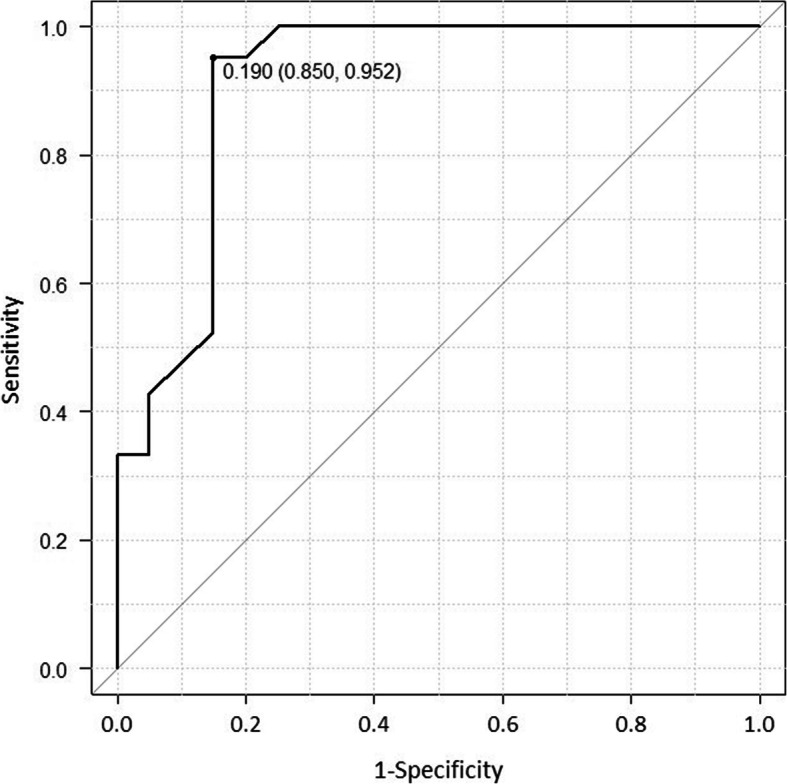
A receiver-operating characteristic (ROC) curve to identify the cut-off value of the T1 pelvic angle divided by pelvic incidence for predicting disability at 2 years post-operatively. The area under the ROC curve was 0.88 (95% confidence interval, 0.850–0.952)

The diagnostic performance of pre-operative T1PA/PI values was assessed using the ROC curve. The cut-off value of > 0.19 had a sensitivity of 95% and specificity of 85% (Fig. 4). Even though there was no statistical difference between the two groups pre-operatively, except for body height (*p* = 0.021), the post-operative VAS-satisfaction (*p* < 0.001), HOOS-JR (*p* = 0.023), and EuroQol 5-Dimension 5-Level (*p* = 0.041) differed when the pre-operative T1PA/PI cut-off value was chosen as 0.2 (Table 3).

**Table 3:** Between-group comparisons of the pre-existence of global sagittal deformity

	T1 pelvic angle divided by pelvic incidence		
	> 0.2 *n* = 36	≤0.2 *n* = 172	*P* value
Age (years)	74.1 ± 6.7	74.3 ± 7.1	0.653
Male (n, *%*)	5, *13.9*	20, *11.6*	0.704
Body height (cm)	154.1 ± 3.9	156.6 ± 3.3	0.021*
Body mass index (kg/m^2^)	24.2 ± 3.6	24.7 ± 2.9	0.548
Visual analogue scale-satisfaction (mm) ^a, b^	76.1 ± 17.7	76.5 ± 19.4	0.999
	27.2 ± 20.4	19.5 ± 18.3	< 0.001*
HOOS-JR ^b^	46.1 ± 12.9	46.2 ± 15.2	0.773
	71.2 ± 11.7	84.9 ± 12.6	0.023*
EuroQol 5-Dimension ^b^	0.42 ± 0.13	0.43 ± 0.12	0.564
	0.66 ± 0.11	0.70 ± 0.12	0.041*

Data are expressed as mean ± standard deviation values or the number of hip involvements (*%*) as appropriate for the data type

*P < .05; represents significant between-group differences

*HOOS-JR* the Hip Disability and Osteoarthritis Outcome Score Joint Replacement

^a^Patient satisfaction after THA evaluated using a 100-mm VAS for satisfaction with anchors at “0” mm (complete satisfaction) and “100” mm (complete dissatisfaction)

^b^Between-group comparisons of outcomes pre-operatively (upper row) and at the 2-year follow-up (lower row)

The reliability in measurement was good (intra-class correlation coefficient [ICC], 0.5–0.75) to excellent (ICC > 0.75). The inter-observer agreement was higher for T1PA than for PI, SVA, and LL measurement (Table 4). The intra- and inter-rater agreements, with a discrepancy of < 2°, were as follows: LL, 78.2 and 84.5%; PI, 81.3 and 77.5%; and T1PA, 86.5 and 87.1%, respectively.

**Table 4:** Intra- and inter-observer reliability of the sagittal spinopelvic parameters evaluated using intra- and inter-class correlation coefficients

	C7 sagittal vertical axis	Lumbar lordosis	Pelvic incidence	T1 pelvic angle
Intra-class Correlation Coefficient				
Observer 1	0.71 (0.61–0.83)	0.72 (0.58–0.81)	0.74 (0.61–0.84)	0.77 (0.61–0.85)
Observer 2	0.67 (0.52–0.82)	0.69 (0.62–0.84)	0.73 (0.67–0.86)	0.76 (0.64–0.83)
Observer 3	0.70 (0.64–0.82)	0.71 (0.65–0.87)	0.72 (0.64–0.87)	0.78 (0.63–0.82)
Inter-class Correlation Coefficient				
	0.67 (0.55–0.76)	0.63 (0.53–0.74)	0.74 (0.58–0.83)	0.80 (0.61–0.84)

Values are given as coefficients with a corresponding 95% confidence interval in parentheses

## Discussion

The most important finding of our study was that the pre-existence of global sagittal deformity was associated with patient disability after THA at the 2-year follow-up (*p* = 0.010) (Table 1). Clinicians should be aware that a spinal sagittal deformity might lead to poor patient-reported outcomes after THA, particularly among patients with a T1PA/PI > 0.2 and/or a short stature (Table 2).

Previous studies that have evaluated sagittal spinopelvic parameters on THA outcomes have employed dislocation and revision as the study end-points [4, 9, 11]. Other studies have focused on evaluating measures of alignment obtained in sitting and standing postures as dynamic risk factors for dislocation [8, 10]. Only a few studies have retrospectively evaluated the effect of the pre-operative sagittal spinopelvic alignment on outcomes after THA [23, 30, 31]. Ochi et al. found that THA patients with a pre-operatively imbalanced sagittal alignment had poorer outcomes according to the modified HHS and that pre-operative spinopelvic alignment predicted post-operative hip function at 3 to 26 months [23]. Perrone et al. proposed that patients with a high PI had a significantly better HOOS after THA than those with a low PI (56.4° versus 48.7°, *p* = 0.006) [30]. These studies did not evaluate the relationship between global sagittal deformity and functional disability after THA.

In our study, we used T1PA/PI measures to evaluate the effects of global sagittal deformity on patient disability after THA. The T1PA combines information from both the SVA and pelvic tilt simultaneously to measure the geometry of global spinal deformity more directly [6]. Our results showed that a pre-operative T1PA/PI > 0.2 was associated with lower satisfaction after THA (*p* < 0.001) (Table 3). Moreover, body height < 148 cm (*p* = 0.011) was an independent risk factor for persistent disability (Table 2). A short stature, defined by a body height of < 148 cm equivalent to legal standards, can lead to an atypical load distribution on the spine and a delay in the process of ossification [13]. The proportion of patients with a short stature in our study group was higher than the 0.8% rate reported by Anis et al. [13] (Table 1). We did identify that patients with a T1PA/PI > 0.2 were shorter than the others (*p* = 0.021; Table 3). Moreover, there was no difference between the two groups with respect to the radiographic severity of PI or PT (Fig. 3).

This study had several limitations. The main limitation was the relatively small study sample, which limited the statistical power of our results. Second, we investigated only cemented prostheses implanted using a direct lateral approach [21, 22]. It may be difficult to apply our results to other populations. We do note that, among Asian populations, the primary indication for THA is secondary OA caused by developmental acetabular dysplasia, with a greater prevalence in women than in men [22, 23]. In fact, only 12.0 and 8.7% of our patients were men and primary hip OA, respectively; therefore, our results cannot be generalised to other implant types, approaches, or ethnicities [4, 12, 32] (Table 1). Third, the analyses cannot be performed for dynamic changes with the patient in the sitting or supine position [7]. Lastly, our follow-up period was relatively short [14, 16]. Additional follow-up information would be required to determine long-term results [14].

Despite these limitations, our study does highlight that several pre-operative factors could affect functional disability 2 years after THA. It could be that their post-operative satisfaction merely reflects general personalities and/or medical expectations, rather than being a proxy for recovery, among other things; however, the strength of our study lies in the finding that the pre-operative T1PA/PI was associated with disability after THA. Our findings are clinically relevant and indicate that spinopelvic sagittal alignment should be precisely evaluated before THA to improve patient satisfaction [11]. Sagittal alignment measurements would also help explain potential differences in PI and in compensation and may clarify the severity of OA.

The management of these individuals could include perioperative interventions, such as the prescription of an orthosis and/or physical therapy, or involve prediction of subsequent spinal surgery. In our findings, the focus on PROMs also provides novel information on possible differences among patients with and without a T1PA/PI > 0.2; this could be helpful in setting expectations for patients and surgeons before THA (Table 4). Interestingly, the thresholds obtained from the ROC curve in this study was similar to that reported in a previous study [6] (Fig. 4). To the best of our knowledge, this is the first study about the relationship between spinopelvic alignment and PROMS after THA.

## Conclusion

Global sagittal deformity, especially in patients with a T1PA/PI > 0.2 and/or short patient stature, was associated with a higher disability rate at the 2-year follow-up after THA. Clinicians should be aware of the influence of several pre-operative factors on disability, 2 years after THA. Further studies are warranted to improve our understanding of PROMs, long-term function, and patient satisfaction after THA.

## Data Availability

The datasets used and/or analysed during the current study are available from the corresponding author on reasonable request.

## Abbreviations

CI: Confidence interval
HHS: Harris hip score
HOOS-JR: Hip disability and osteoarthritis outcome score-joint replacement
ICC: Intra-class correlation coefficient
LL: Lumbar lordosis
OA: Osteoarthritis
OR: Odds ratio
PA: Pelvic angle
PI: Pelvic incidence
PT: Pelvic tilt
PROM: Patient-reported outcome measures
SVA: C7 sagittal vertical axis
THA: Total hip arthroplasty
VAS: Visual analogue scale
